# Biological control potential of worrisome wheat blast disease by the seed endophytic bacilli

**DOI:** 10.3389/fmicb.2024.1336515

**Published:** 2024-03-11

**Authors:** Musrat Zahan Surovy, Sudipta Dutta, Nur Uddin Mahmud, Dipali Rani Gupta, Tarin Farhana, Sanjay Kumar Paul, Joe Win, Christopher Dunlap, Ricardo Oliva, Mahfuzur Rahman, Andrew G. Sharpe, Tofazzal Islam

**Affiliations:** ^1^Institute of Biotechnology and Genetic Engineering (IBGE), Bangabandhu Sheikh Mujibur Rahman Agricultural University, Gazipur, Bangladesh; ^2^The Sainsbury Laboratory, Norwich Research Park, Norwich, United Kingdom; ^3^Crop Bioprotection Unit, National Center for Agricultural Utilization Research, Agricultural Research Service, United States Department of Agriculture (USDA), Peoria, IL, United States; ^4^World Vegetable Center, Shanhua, Taiwan; ^5^W.V.U. Extension Service, West Virginia University, Morgantown, WV, United States; ^6^Global Institute for Food Security, Saskatoon, SK, Canada

**Keywords:** *bacillus*, antagonist, blast severity, genome, antimicrobial defense, antibiosis

## Abstract

Crop production often faces challenges from plant diseases, and biological control emerges as an effective, environmentally friendly, cost-effective, and sustainable alternative to chemical control. Wheat blast disease caused by fungal pathogen *Magnaporthe oryzae Triticum* (MoT), is a potential catastrophic threat to global food security. This study aimed to identify potential bacterial isolates from rice and wheat seeds with inhibitory effects against MoT. In dual culture and seedling assays, three bacterial isolates (BTS-3, BTS-4, and BTLK6A) demonstrated effective suppression of MoT growth and reduced wheat blast severity when artificially inoculated at the seedling stage. Genome phylogeny identified these isolates as *Bacillus subtilis* (BTS-3) and *B. velezensis* (BTS-4 and BTLK6A). Whole-genome analysis revealed the presence of genes responsible for controlling MoT through antimicrobial defense, antioxidant defense, cell wall degradation, and induced systemic resistance (ISR). Taken together, our results suggest that the suppression of wheat blast disease by seed endophytic *B. subtilis* (BTS-3) and *B. velezensis* (BTS-4 and BTLK6A) is liked with antibiosis and induced systemic resistance to wheat plants. A further field validation is needed before recommending these endophytic bacteria for biological control of wheat blast.

## 1 Introduction

Wheat (*Triticum aestivum* L.) is the most commonly grown cereal crop worldwide and a major source of protein and calories for people, providing between 20 and 25% of needs (Chakraborty et al., 2020). It is the main nutrient source for 40% of the world’s population (Giraldo et al., 2019). The consumption of wheat has been steadily increasing year over year. Among several biotic and abiotic stressors, wheat blast disease caused by a lineage of *Magnaporthe oryzae* is a devastating disease, which poses serious threat to wheat production in several countries in South America (Brazil, Argentina, Bolivia and Paraguay), Asia (Bangladesh) and Africa (Zambia) (Islam et al., 2020). First emerged in Brazil in 1985, the wheat blast was gradually spread to neighboring Argentina, Bolivia and Paraguay (Islam et al., 2019). In February 2016, Bangladesh faced the wheat blast for the first time in a country outside of South America and devastated more than 15,000 hectares of wheat fields with up to 100% yield losses (Islam et al., 2016). It was also identified in Zambia very recently (Tembo et al., 2020) and potentially threatens wheat production in Europe (Barragan et al., 2022). Genomic analysis revealed that both Bangladesh and Zambian wheat blast was caused by an aggressive clonal population of a South American lineage of *M. oryzae* (anamorph *Pyricularia oryzae*) *Triticum* (MoT) (Latorre and Vincent, 2023). These findings created a worldwide concern that this deadly wheat killer may spread to neighboring countries of Bangladesh, India and China that are ranked world’s second and first in wheat production, respectively.

MoT, a filamentous and heterothallic ascomycete fungus, can infect more than 50 grass species (Ceresini et al., 2019). Immediately after infection, it blocks the vascular system, resulting white head symptoms (Ceresini et al., 2019). Application of chemical fungicides after head blast appearance is ineffective. Moreover, high reliance on chemical fungicides is ineffective and also negatively impacts the environment, soil, and human health. Fungicide treatments are expensive to resource poor farmers and poses the risk of resistance development if used recurrently (Poloni et al., 2021). The development of resistant varieties against wheat blast faces an uphill battle due to the scarcity of resistance genes identified so far (Wang et al., 2018). In addition, plant resistance is likely to be less durable in the field due to the evolution of new MoT races (Figueroa et al., 2018). Therefore, the development of an effective biological control agent (BCA) together with other bio-rational options may be an efficient approach to control MoT.

Plant-beneficial microorganisms such as bacteria can be attractive natural alternatives to chemicals for biological control of destructive phytopathogens such as MoT (Surovy and Islam, 2021). Isolation of untapped beneficial bacteria from particular environments, such as internal plant tissues, is a new research approach (Khan et al., 2016). Bacteria colonize inner plant tissues for all or part of their lifetime and promote the growth and fitness of host plants against biotic and abiotic stresses (Hardoim et al., 2015). Like gut microflora in humans, bacteria inside plants exhibit complex interactions with their hosts and have been proven as a potential source of biocontrol agents (Surovy and Islam, 2021). Numerous studies suggest that beneficial bacteria can directly inhibit pathogenic growth by producing various primary (Surovy et al., 2019; Surovy and Islam, 2021) and secondary metabolites (Heenan-Daly et al., 2021) or inducing host systemic resistance (ISR) (Singh et al., 2021). They also promote host growth through solubilization of nutrients (Lacava et al., 2021; Mahmud et al., 2021), nitrogen fixation (Islam et al., 2016), and production of plant growth regulators (Khan et al., 2017). Previous studies suggest that certain groups of beneficial bacteria can protect rice plants from blast fungus (Chen et al., 2020), and bacterial species under the genera of *Serratia*, *Pseudomonas* (Arriel-Elias et al., 2021; Patel et al., 2021), *Streptomyces* (Awla, 2021), *Burkholderia* (Lin et al., 2021), and *Bacillus* (Lam et al., 2021) have been reported as effective antagonists for biological control of rice blast fungus. There are only a few reports describing that the pure compounds from some bacteria can suppress the growth of MoT *in vitro* (Chakraborty et al., 2020; Paul et al., 2022). However, no reports have been published on the biological control of wheat blast by beneficial bacteria from rice and wheat seeds.

To develop effective biologicals to control wheat blast, the specific objectives of the study were to - (i) screen bacterial antagonists to MoT from the seeds of local cultivars of wheat and rice grown in Bangladesh; (ii) evaluate the inhibitory effects of selected bacterial isolates against MoT *in vivo*; (iii) identify the potential bacterial isolates through genome sequencing; and (iv) elucidate the underlying molecular mechanisms of bacterial isolates to control wheat blast.

## 2 Results

### 2.1 Seed endophytic bacteria suppress mycelial growth of MoT

#### 2.1.1 Dual culture assay

**Figure 1:**
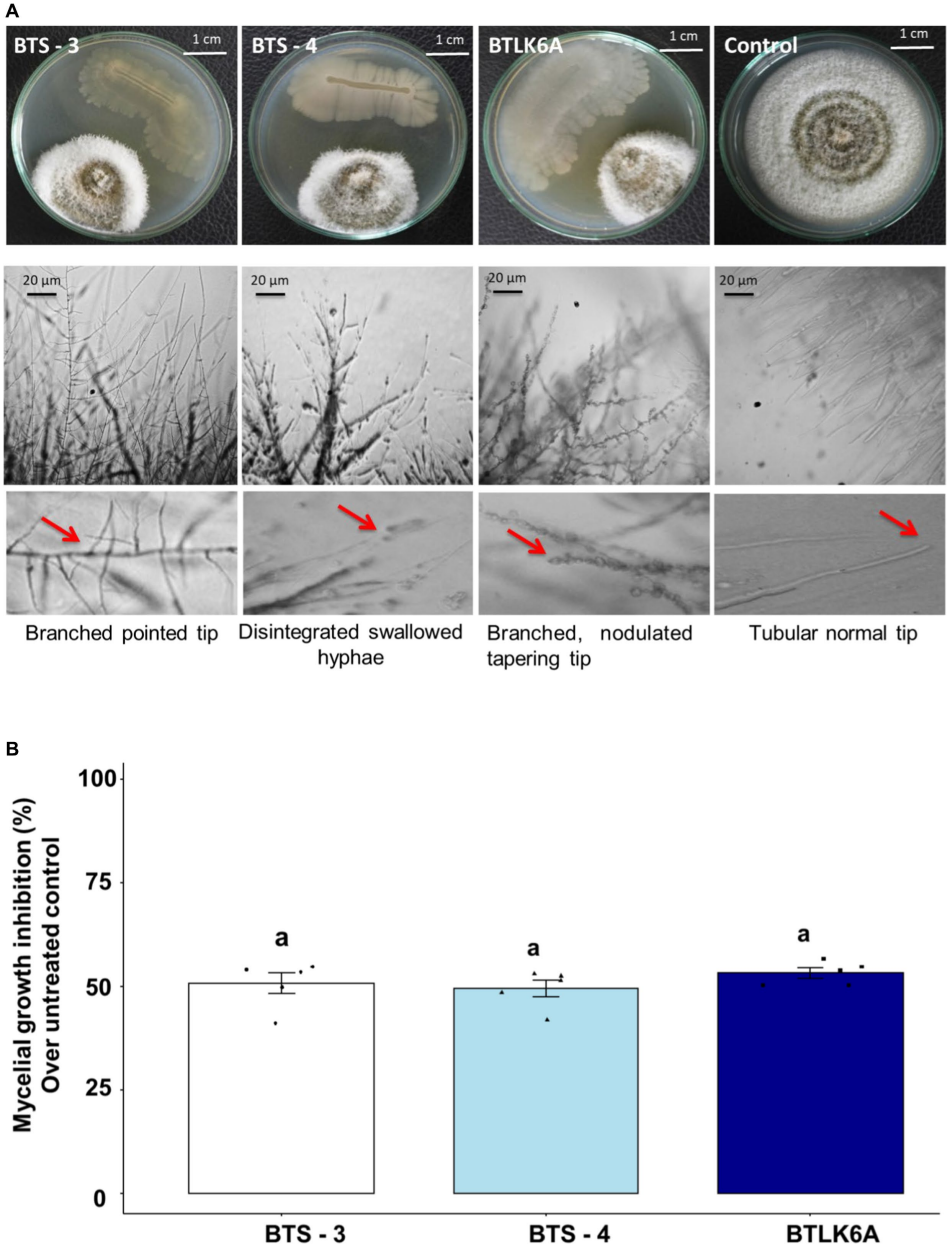
Inhibitory effects of bacterial isolates on mycelial growth of MoT. **(A)** Micrograph representing the suppression of mycelial growth and induction of morphological alternations in MoT hyphae approaching the colonies of bacterial isolates in PDA; **(B)** MoT mycelial growth inhibition (%) by bacterial isolates over untreated control. Data were recorded 14 days after inoculation and incubated at 25(±2) °C (*n* = 5, ANOVA with Tukey multiple comparison test, *p* ≤ 0.05; same letters on bars are not statistically significantly varied from each other). The black dots represent the data points for each bacterial isolate.

Three seed endophytic bacterial isolates *viz.* BTS-3, BTS-4, and BTLK6A were identified as potential biocontrol agents against MoT in dual culture assay from the screening of 170 isolates (Supplementary Table S1) (Figure 1). All three bacterial isolates showed strong but differential inhibition of MoT hyphal growth (Figure 1A). The MoT inhibition rate by different bacterial isolates significantly varied from untreated control (F3, 16 = 217.25, *p* ≤ 0.001). The inhibition of MoT mycelial growth varied from 49.42 to 53.16%, with the highest mycelial inhibition observed in BTLK6A (53.16%), followed by BTS-3 (50.71%) and BTS-4 (49.42%) (Figure 1B). Microscopic analyses showed that the untreated control MoT had regularly branched hyaline tubular hyphae with smooth and intact structures (Figure 1A). However, BTS-3 bacterial treatment induced excessive branching with pointed tips. BTS-4 induced swelling and disintegration of MoT hyphae along with excessive branching. Nodulation with hyper branching and tapering of MoT hyphal tips were found in MoT mycelia treated with BTLK6A bacterial isolate (Figure 1A).

#### 2.1.2 Cell-free culture filtrate of bacterial isolates suppresses mycelial growth of MoT

**Figure 2:**
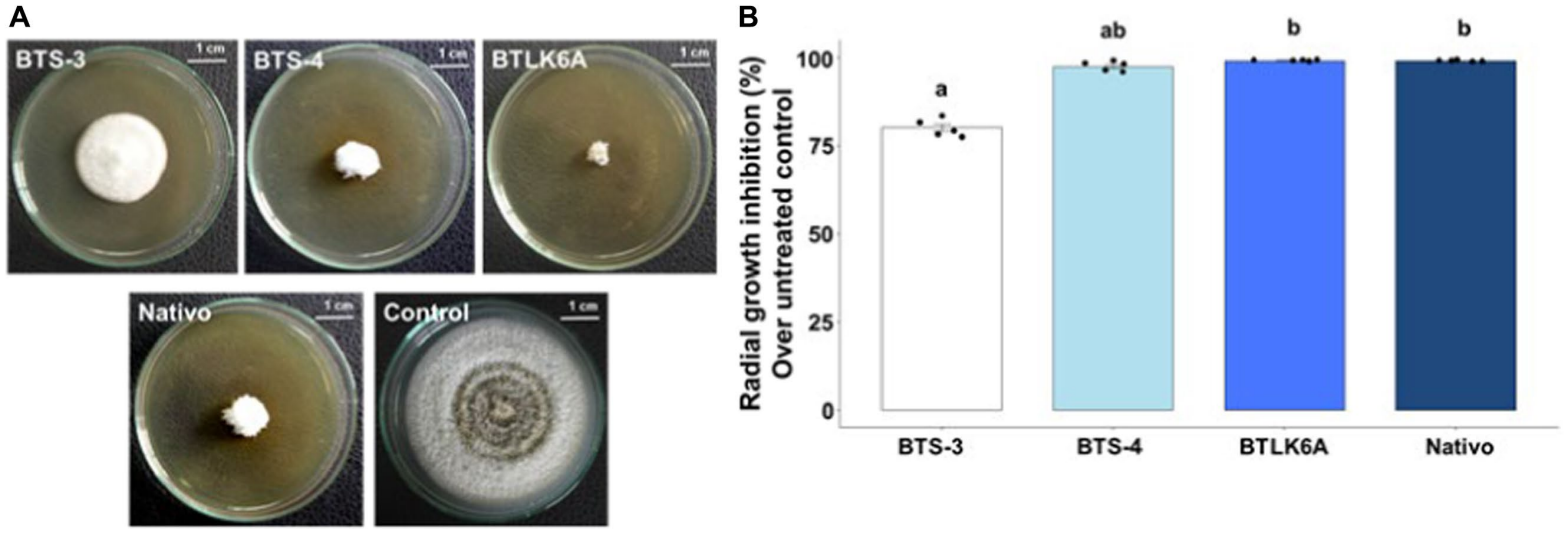
Inhibitory effects of cell-free autoclaved bacterial culture filtrates on MoT mycelial growth. **(A)** Inhibitory effects of bacterial cell-free culture filtrates on mycelial growth of MoT in PDA plates; **(B)** Inhibition of MoT mycelial growth (%) by bacterial cell-free culture filtrates over untreated control. Data were recorded 14 days after inoculation and incubated at 25 (±2) °C (*n* = 5, Kruskal-Wallis test with Dunn multiple comparison test, chi-squared value = 22.3, *p* ≤ 0.001; same letters on bars are not statistically significantly varied from each other). Black dots represent the data points for each bacterial isolate.

The effect of cell-free autoclaved bacterial filtrates was tested to determine whether the antifungal activity of the bacterial isolates was due to the secretion of antifungal compounds in the liquid culture media. The bacterial cell-free culture filtrates showed significant suppression of the radial growth of MoT (Figure 2A). The lowest radial growth [0.49 cm] was recorded in the culture plate containing the cell-free culture filtrate of BTLK6A, which was statistically non-significant (99.10%) to the radial growth inhibition by the commercial fungicide Nativo (60 ppm) (*p* = 0.73). However, 97.56% [1.45 cm] inhibition of MoT radial mycelial growth was recorded for BTS-4 treatment, and 80.19% [11.79 cm] reduction was recorded for BTS-3 compared to the untreated control (Figure 2B).

### 2.2 Assessment of biocontrol effects of bacteria on seedling assay

**Figure 3:**
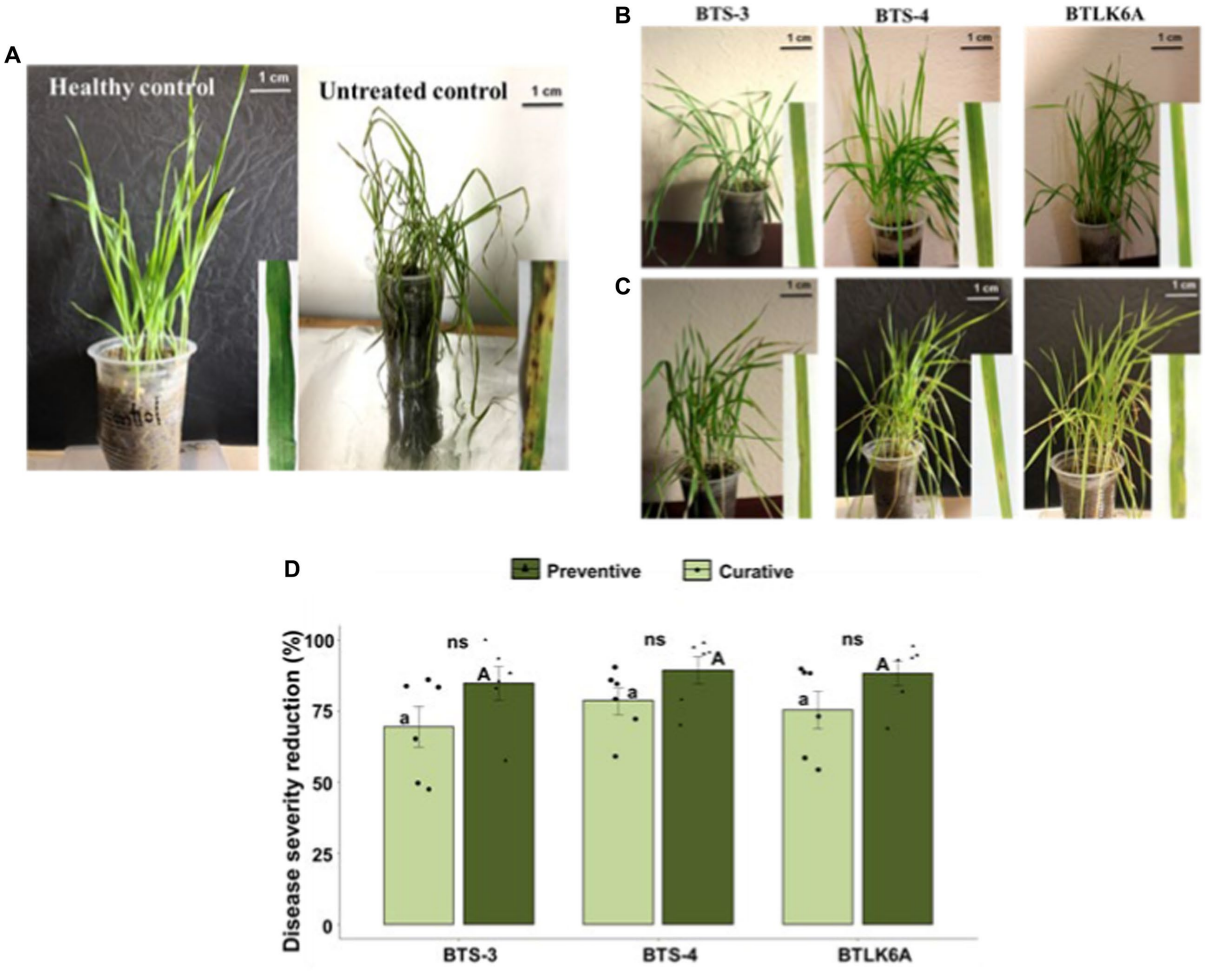
Suppression of leaf blast disease in wheat seedlings cv. BARI Gom-24 (Prodip) by bacterial isolates. **(A)** Seedlings of healthy control (no bacteria or MoT inoculation) and untreated control (plants only inoculated with MoT); **(B)** Effects of bacterial preventive control measure to control seedling leaf blast caused by MoT; **(C)** Effects of bacterial curative control measure to control leaf blast caused by MoT; **(D)** Reduction of leaf blast disease severity (%) in wheat seedlings by application of bacterial isolates compared to untreated control. As a preventive control measure, wheat seedlings were sprayed with bacterial isolates 24 h prior to the inoculation of MoT conidia, whereas wheat seedlings were sprayed with bacterial isolates 24 h after MoT conidia in curative control measure. Same letters on bars are not significantly different from each other; small letters on bars represent significant differences among curative bacterial control measures, and capital letters on bars represent significant differences among preventive bacterial control measures. Non-significant differences (ns) were observed in curative and preventive control measures of each bacterial isolate (ANOVA with Tukey multiple comparison test, *n* = 6, *p* ≤ 0.05, each pot contained 10 seedlings and treated as one replicate).

A pot experiment was performed to evaluate the *in vivo* efficacy of bacterial isolates (BTS-3, BTS-4, and BTLK6A) against MoT. Eye-shaped blast symptoms were observed 3–4 days after MoT inoculation in both treatments pertaining to preventive and curative control measures. A significant variation in the biocontrol efficacy of each bacterial isolate was observed on seedlings under both control measures compared to untreated control. Furthermore, seedlings inoculated with bacterial isolates as preventive measures demonstrated lower disease severity than curative control, which is consistent with any biological control measure. The reduction of seedling blast severity ranged from 84.66 to 89.31% in preventive control measures by bacterial isolates. The highest reduction of seedling disease severity was recorded in BTS-4 (89.31%), followed by BTLK6A (88.25%) and BTS-3 (84.66%) (Figures 3A–C). However, the differences are statistically non-significant (*p* = 0.472) (Figure 3D). In curative control measures, the reduction of seedling disease severity ranged from 69.45 to 78.54%. Similar to preventive control, the highest reduction was documented for BTS-4 (78.54%), followed by BTLK6A (75.35%) and BTS-3 (69.45%) (Figure 3D).

### 2.3 Identification of potential bacterial isolates

#### 2.3.1 Morphological and physiological characterization of bacterial isolates

All three bacterial isolates were creamy white in color, oxidase and catalase positive, motile, and grew well on NaCl-supplemented NBA medium. The BTS-4 isolate showed the highest salt tolerance and grew well up to 10% NaCl concentration. However, only the BTLK6A was able to produce indole-3-acetic acid (Table 1).

**Table 1:** Morphological and physiological characterization of three potential bacterial isolates against MoT.

Bacterial isolate	Source	Colony characteristic	Biochemical test										IAA
			KOH	Gram	Catalase	Oxidase	Motility	Salinity tolerance (%)					
								2	4	6	8	10	
BTS-3	Rice seeds cv. Rangabinni	White, sticky, round	−	+	+	+	+	+	+	+	+	−	−
BTS-4		White, sticky, round	−	+	+	+	+	+	+	+	+	+	−
BTLK6A	Wheat seeds cv. Kanchan	White, irregular	−	+	+	+	+	+	+	+	−	−	+

#### 2.3.2 Molecular identification of bacterial isolates

**Figure 4:**
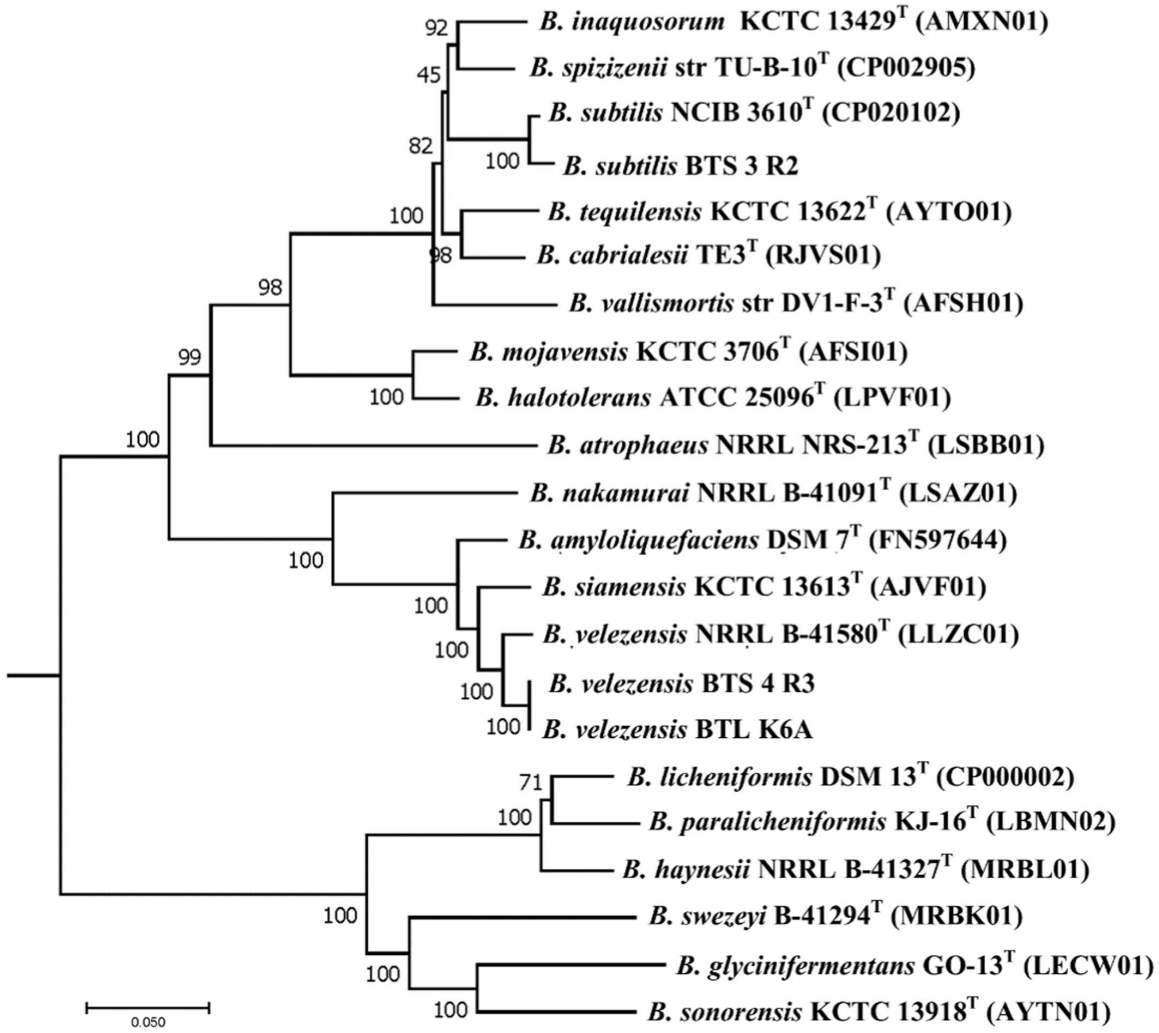
Phylogenetic neighbor-joining tree reconstructed from the core genomes of selected type strains of species from the *Bacillus subtilis* group. Bootstrap values 50%, based on 1,500 pseudoreplicates are indicated on branch points. *B. indicus* was used as an out group, and only the relevant part of the tree is presented. The scale bar corresponds to 0.05 nucleotide substitutions per site.

Earlier the three selected bacterial isolates were identified as *Bacillus subtilis* BTS-3, *B. amyloliquefaciens* BTS-4, and *B. amyloliquefaciens* BTLK6A based on 16S rRNA gene sequencing (Chanclud et al., 2017; Surovy et al., 2017; Dutta et al., 2018). Changes on the genomic levels due to the evolution of plant-associated life cycle, reinvestigation of the taxonomic status of our bacterial isolates was performed following the phylogenomic analysis proposed by Dunlap et al. (2016), and later they were identified as *Bacillus subtilis* BTS-3 (NCBI accession WOVJ00000000), *B. velezensis* BTS–4 (accession number WOVK00000000), and *B. velezensis* BTLK6A (accession number WOYD00000000). The phylogenetic relationships of the selected bacterial isolates (Supplementary Table S2) are shown in Figure 4. The phylogenetic tree revealed that BTS-4 and BTLK6A are closely related and distributed at the same node. At the same time, BTS-3 is distantly related to BTS-4 and BTLK6A.

### 2.4 Whole genome sequencing reveals the general genomic features of antagonistic bacteria

The *de novo* assembly of BTS-3 estimated 4,121,943 bp chromosome size with 47 contigs. The overall G + C content of the assembly was 43.5%, with an N 50 of 1,063,829 and an L 50 value of 2. The genome analysis predicted 117 RNA genes and 4,272 protein-coding genes in putative functional categories. The largest contig assembled was 1,140,720 bp, and the average coding sequence size was 849 bp (Table 2). The estimated genome sizes of BTS-4 and BTLK6A were 3,907,662 bp and 3,908,699 bp, respectively, with 27 and 32 contigs in each assembly. The N 50 values of the assemblies were 2,032,688 and 1,024,542, whereas the L 50 assembly values were 1 and 2, respectively. Both genomes predicted 113 RNA genes and protein-coding genes (3,966 in BTS-4 and 3,968 in BTLK6A). The largest contigs assembled for BTS-4 and BTLK6A were 2,032,688 and 1,083,238 bp, respectively, with an average coding sequence size of 881 and 880, respectively (Table 2).

**Table 2:** General genomic features of bacterial isolates.

Genomic features	BTS-3	BTS-4	BTLK6A
Closest strain	*Bacillus subtilis*	*Bacillus velezensis*	*Bacillus velezensis*
Genome size (bp)	4,121,943	3,907,662	3,908,699
Contigs	47	27	32
Largest contigs	1,140,720	2,032,688	1,083,238
G + C content (mol%)	43.5	46.5	46.5
N 50 (bp)	1,063,829	2,032,688	1,024,542
L 50	2	1	2
Protein-coding sequences	4,272	3,966	3,968
Percent of coding region	88.0	89.4	89.3
Average CDS size (bp)	849	881	880
Total number of RNAs	117	113	113
Number of Ribosomal RNAs	17	14	17
Number of tRNAs	85	84	84
Phage-associated genes	13	13	13
Number of Subsystems	477	463	463

#### 2.4.1 Subsystem analysis of bacterial isolates

**Figure 5:**
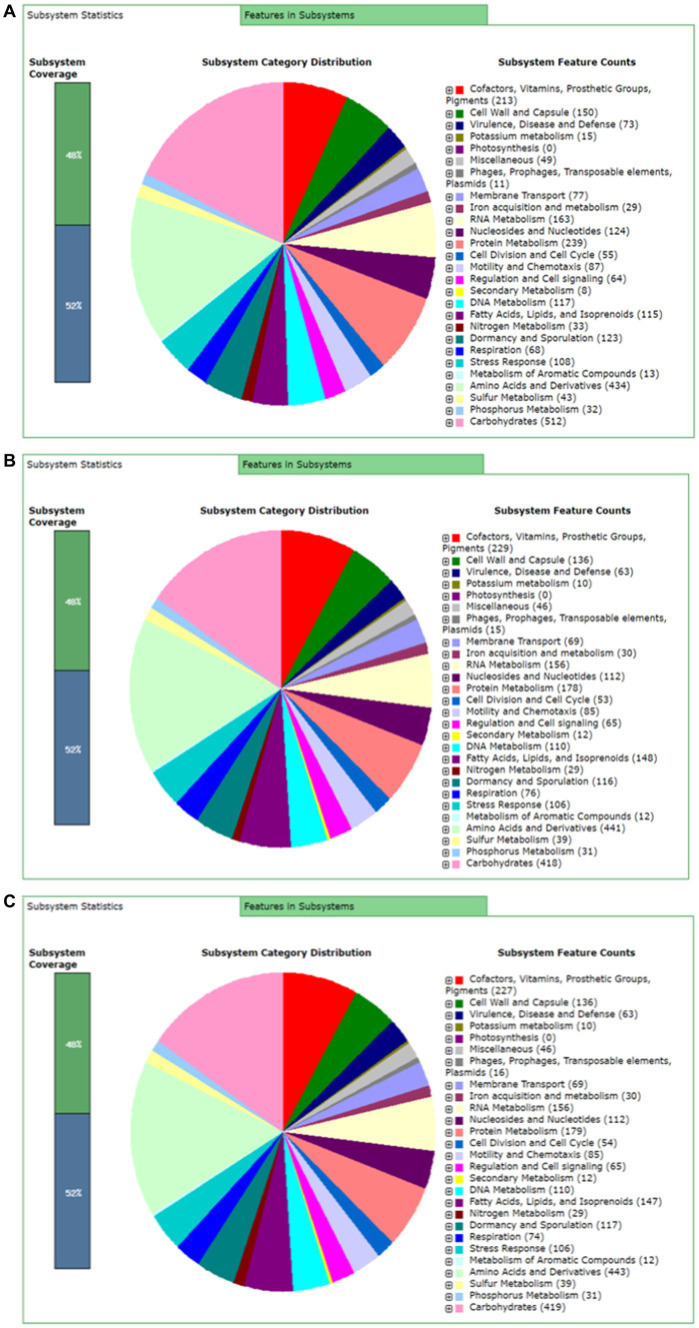
RAST server predicted subsystem categories for bacterial isolates. **(A)** Subsystem categories for *Bacillus subtilis* BTS-3; **(B)** Subsystem categories for *Bacillus velezensis* BTS-4; and **(C)** Subsystem categories for *Bacillus velezensis* BTLK6A.

Rapid Annotation using subsystem technology (RAST) predicted 477 subsystems for BTS-3 bacterial isolate. Among those, 73 subsystems are responsible for virulence, disease and defense; 87 for motility and chemotaxis; 8 for secondary metabolism; 29 for iron acquisition and metabolism; and 64 for regulation and cell signaling (Figure 5A). Both BTS-4 and BTLK6A predicted 463 subsystems, with 63 subsystems accountable for virulence, disease and defense; 85 for motility and chemotaxis; 12 for secondary metabolism; 30 for iron acquisition and metabolism and 65 for regulation and cell signaling (Figures 5B, C).

#### 2.4.2 Bacterial genomic features for antagonism

**Figure 6:**
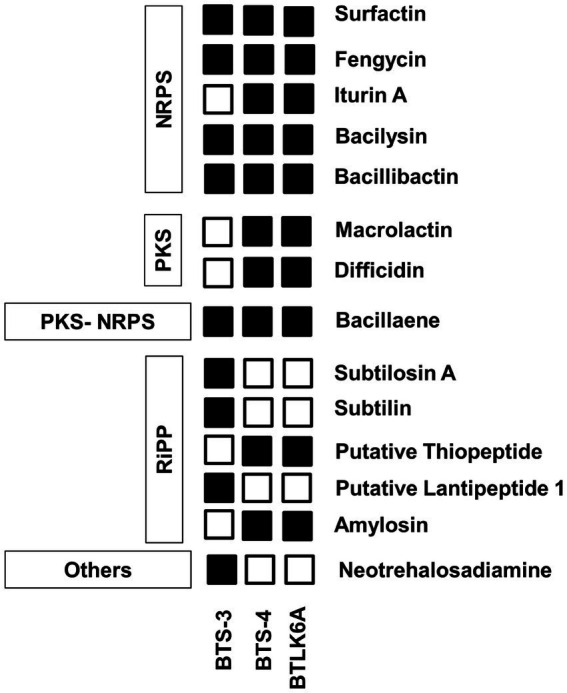
Identified features of bacterial antimicrobial peptides for antagonism. PKS, polyketide synthase; NRPS, Nonribosomal peptide synthetases; RiPP, Ribosomally synthesized and post-translationally modified peptide.

The BTS-3 genome encodes several orthologs of intrinsic genes related to antimicrobial peptides, such as bacillaene (*baeBCDEGHIJLMN*), bacilysin (*bacABCDEFG*), bacillibactin (*dhbABCE*), and fengycin (*fenABCDE*) (Table 3; Figure 6). Additionally, it harbors several gene clusters associated with antioxidant defense enzymes like-superoxide dismutase (*sodACF*), glutathione peroxidise (*bsaA*), catalase (*katAEX*), and thiol peroxidase (*Tpx*). Moreover, the genome contains cell wall degrading gene clusters, including esterase (*estAB*), endoglicanase (*eglS*), beta-glucanase (*bglS*), and pectatelyase (*pelABC*). It also encodes for the gene cluster related to the volatile compound acetoin (*alsSD*) (Table 3).

**Table 3:** Gene clusters identified in BTS-3, BTS-4, and BTLK6A bacterial genome.

Compound	Enzyme	BTS-3	BTS-4	BTLK6A
Gene clusters related to antibiotic production				
Bacillaene	PKS/NRPS	*baeBCDEGHIJLMN*	*baeBCDEGHIJLMN*	*baeBCDEGHIJLMN*
Macrolactin	PKS	Not present	*mlnABCDEFGHI*	*mlnABCDEFGHI*
Difficidin	PKS	Not present	*dfnABCEFGHIJKLM*	*dfnABCEFGHIJKLM*
Bacilysin(siderophore)	NRPS	*bacABCDEFG*	*bacABCDEFG*	*bacABCDEFG*
Bacillibactin	NRPS	*dhbABCE*	*dhbABE*	*dhbABCE*
Fengycin	*NRPS*	*fenABCDE*	*fenABCDE*	*fenABCDE*
Iturin A	NRPS	Not present	*ituA*	*ituA*
Gene clusters related to antioxidant enzyme				
Superoxide dismutase		sodACF	sodACF	sodACF
Glutathione peroxidise		*bsaA*	*bsaA*	*bsaA*
Catalase		*katAEX*	*katAEX*	*katAEX*
Thiol peroxidase		*Tpx*	*Tpx*	*Tpx*
Gene cluster related to cell wall degradation				
Esterase		*estAB*	*estAB*	*estAB*
Endoglucanase		*eglS*	*eglS*	*eglS*
Beta-glucanase		*bglS*	*bglS*	*bglS*
Pectatelyase		*pelABC*	*pelAB*	*pelAB*
Gene cluster related to volatile compound				
Acetoin		*alsSD*	*alsSD*	*alsSD*

BTS-4 and BTLK6A have same type of intrinsic genes of the antimicrobial peptide to BTS-3, with the additional Iturin A (*ituABCD*), macrolactin (*mlABCDEFGHI*), and difficidin (*dfnABCDEFGHIJKLM*) genes (Table 3; Figure 6). The gene cluster related to antioxidant defense enzyme, cell wall degradation enzyme, and volatile compounds in BTS-4 and BTLK6A were matched to those of BTS-3 (Table 3).

## 3 Discussion

The invasive wheat blast fungus is a new serious threat to the food and nutritional security of Bangladesh, India, and other Asian wheat-growing regions (Tembo et al., 2020). Traditional approaches for controlling this troublesome disease are ineffective, and breeding resistant varieties take several years. A blast resistance genetic resource, the *2NS* chromosomal segment, has been used in South America and even in Bangladesh against MoT fungus. However, resistance to wheat blast conferred by the *2NS* translocation has been overridden by some strains of MoT in Bolivia. This study attempted a novel strategy to develop a biorational management strategy for managing MoT using beneficial bacteria from wheat and rice seeds that are locally available.

*In vitro* dual culture assay displayed that certain bacterial strains isolated from seeds have a consistent ability to inhibit the growth of wheat blast fungus (MoT). Three bacterial isolates (BTS-3, BTS-4, and BTLK6A) showed variable inhibitory activity against MoT. Notably, those bacterial isolates caused morphological alternations to the MoT hyphae, such as excessive branching, swelling, and cell disintegration in the approaching hyphae. The probable mechanisms behind the bacterial antagonism to MoT growth may include the production of secondary metabolites, biofilm formation, secretion of lytic or cell wall degrading enzymes, and competition for resources (space or nutritions) (Lam et al., 2021; Surovy and Islam, 2021). The results of the autoclaved cell-free culture filtrate assay indicate that the bacteria isolates tested are capable of producing heat-stable extracellular antimicrobial compounds that can inhibit the growth of MoT on nutrient agar medium (PDA). According to a study by Chakraborty et al (2020), linear lipopeptides from marine *B. subtilis* and oligomycins from *Streptomyces* spp. were found to be effective in controlling MoT. However, there is a lack of literature dealing with the use of rice or wheat-derived bacterial isolates in controlling the wheat blast pathogen, MoT. Many research studies are available on the biological control of rice blast disease by various beneficial bacterial isolates (Lam et al., 2021; Zhu et al., 2021). It has been reported that the heat-stable cell-free culture filtrates from probiotics suppress the mycelial growth of rice blast (Leelasuphakul et al., 2006; Prasanna et al., 2021). Induction of variable hyphal morphological alterations and branching patterns due to disruption of cytoskeletal filamentous actins in hyphae by diverse biocontrol bacterial metabolites has been reported (Islam, 2008; Deora et al., 2010; Islam and Fukushi, 2010). A further study is warranted to characterize the thermostable antimicrobial compounds and their mode of actions in the cell-free culture filtrates that effectively control the growth of MoT fungus.

The success of biocontrol agents largely depends on the survival and shelf-life of the microbial agents. Several lines of evidence suggest that the efficacy of BCAs was higher in the controlled greenhouse and lower in field conditions due to the complex interactions with uncontrolled environmental factors (Zaidi et al., 2014; David et al., 2017). In the current study, the use of bacterial isolates as preventive and curative control measures effectively controlled wheat blast disease in seedlings. In addition, bacterial isolates also enhanced seed germination and growth of wheat seedlings (Supplementary Figure S1; Table 1). This result is consistent with previous research indicating that the bio-inoculation of bacterial isolates can promote plant growth (Rahman et al., 2018). It is worth noting that the seed isolated *Bacillus* spp. used in this study are not harmful to mammals (Surovy et al., 2019; Surovy and Islam, 2021). Application of those bacterial isolates induces genes related to the production of plant growth-promoting hormones (auxin, gibberellin, jasmonic acid, salicylic acid) and also upregulated specific signaling pathways (eg. MAPK signaling pathway) in host plants to promote plant growth (Yan et al., 2022).

Our bacterial isolates were identified as *B. subtilis* BTS-3, *B. amyloliquefaciens* BTS-4, and *B. amyloliquefaciens* BTLK6A initially (Chanclud et al., 2017; Surovy et al., 2017; Dutta et al., 2018). Later, whole genome sequencing identified those isolates as *B. subtilis* BTS-3, *B. velezensis* BTS-4, and *B. velezensis* BTLK6A. Through genome phylogeny, it was revealed that the BTS-4 and BTLK6A bacterial isolates were closely related (Figure 4). Around 318 species consist under the genus *Bacillus* (Gordon et al., 1973); *B. subtilis*, *B. amyloliquefaciens*, *B. pumilus*, and *B. licheniformis* are phenotypically and phylogenetically homogenous and combinedly known as *B. subtilis* species complex (Fritze, 2004). However, many novel *Bacillus* species belonging to the *B. subtilis* species complex have been identified during the last few decades. Phylogenetic analysis using the 16S rRNA gene fails to discriminate all the *B. subtilis* species complex, but complete or whole genome sequencing can distinguish them accordingly (Dunlap et al., 2016).

Additionally, whole-genome mining revealed that all three selected *Bacillus* isolates have gene clusters responsible for biosynthesis of different antimicrobial compounds, cell lytic enzymes, and compounds related to ISR. The major gene clusters identified in the bacterial genomes include macrolactin, bacillaene, bacilysin, bacillibactin, fengycin, and iturin A (Table 3; Figure 6). Biosynthesis and secretion of these compounds by *Bacillus* isolates have been shown direct mechanisms to suppress plant pathogens (Bidima et al., 2022; Wang et al., 2022). Several studies reported that the macrolide compounds such as macrolactin, bacillaene, and difficidin inhibit protein synthesis, impair cell division, and damage the cell membrane to restrict the growth of phytopathogens (Wu et al., 2015; Chen et al., 2021). Moreover, recent studies suggest that these compounds also play a role in transcription regulation and enhance plant resistance against phytopathogens (Chen et al., 2021; Surovy and Islam, 2021). Siderophores such as bacilibactin and bacilysin suppress fungal growth by competing for nutrition (Dimopoulou et al., 2021; Wang et al., 2022).

Interestingly, all three bacterial isolates possess *fenABCDE* gene clusters responsible for fengycin biosynthesis. This compound, also known as plipastatin, has been reported to damage cellular composition and organization by creating vacuoles in hyphae, thus inhibiting fungal growth (Gong et al., 2015). Although three bacterial isolates used in this study possess a diverse range of antimicrobial peptide genes in their genome, more research is needed to investigate which specific compound is precisely effective against MoT.

Gene clusters related to oxidative stress have also been predicted in bacterial genomes. Superoxide dismutase (*soda*, *sodC*, and *sodF*), hydrogen peroxide decomposing catalase (*katA*, *katE*, and *katX*), thiol peroxidase (*tpx*), and glutathione peroxidase (*bsaA*) gene clusters have also been predicted in our selected bacterial isolates. Under biotic and abiotic stress, various reactive oxygen species (ROS) are produced in plant cells and are scavenged or detoxified by various antioxidant enzymes and metabolites. Hasanuzzaman et al. (2022) showed that applying *B. subtilis* can induce coordinated actions of osmoregulation, ion homeostasis, and antioxidant defense in host plants. The presence of these antioxidant enzyme-related gene clusters in our bacterial isolates may indicate that they are involved in ROS metabolism during blast infection.

Plant growth-promoting bacteria can trigger induced systemic resistance (ISR). All three biocontrol bacterial isolates included in this study possessed acetolactate decarboxylase, *alsSD* gene cluster encoding acetoin biosynthesis. Several lines of evidence suggest that acetoin is a powerful elicitor to trigger induced systemic resistance in plants (Peng et al., 2019). Yi et al. (2021) demonstrated that volatile acetoin produced by *B. amyloliquefaciens* UCMB5113 significantly reduces infection of *Bipolaris sorokiniana* and promotes the growth of wheat seedlings compared with seedlings not exposed to bacterial volatiles before pathogen inoculation. The presence of these gene clusters for the biosynthesis of acetoin in our *Bacillus* isolates suggested that they have the potential to induce systemic resistance in wheat plants and suppress blast disease Chowdhury et al. (2015) also showed that the acetoin produced by PGPR is effective in the biocontrol of plant pathogens, and its *in situ* expression takes place during root colonization.

Taken together, current genome analytical data coupled with *in vitro* and *in vivo* bioassays unambiguously suggested that the BTS-3, BTS-4, and BTLK6A have diverse potentials to produce a wide range of antimicrobial compounds, cell wall degrading enzymes and induce systemic resistance to protect wheat plants from MoT. This is the first study demonstrating wheat blast disease suppression by three seed-isolated native *Bacillus* isolates. Findings from the current study have opened a new window for further studies to discover novel clues for the biorational management of wheat blast disease. Therefore, it is necessary to identify and characterize anti-MoT substances produced by these bacterial isolates and elucidate their mechanisms of action. Additionally, large-scale field evaluation using these three potential *Bacillus* isolates (BTS-3, BTS-4, and BTLK6A) at the reproductive stages of wheat are needed for recommending them as candidates for the formulation of biocontrol agents and to design integrated strategies to manage wheat blast disease.

## 4 Materials and methods

### 4.1 Bacterial isolates and growth conditions

Total 170 bacterial isolates were obtained from the bacterial culture collection of the Institute of Biotechnology and Genetic Engineering (IBGE), Bangabandhu Sheikh Mujibur Rahman Agricultural University (BSMRAU), Bangladesh. The pure bacterial cultures were initially isolated from the seeds of traditional rice and wheat cultivars that were preserved in 20% glycerol at −20°C and grown in nutrient broth agar, NBA (25 g in 1 liter, Sigma-Aldrich), for 24–48 h at 25°C. Bacterial suspensions were prepared from the bacterial cultures in nutrient broth (NB) (3 colonies inoculated in 250 mL NB in a 500 mL conical flask) incubated for 48 h in a rotary shaker (120 rpm) at 25°C; cultures were centrifuged (10,000 rpm for 10 min), and the supernatant was discarded. The pellets were subsequently washed 3 times with sterilized distilled water (SDW), and bacterial concentration was adjusted at 1 × 10^8^ (Ceresini et al., 2019) CFU/ml.

### 4.2 Fungal isolate and culture conditions

Wheat blast fungal isolate BTJP-4 (Islam et al., 2016) was also collected from the MoT culture collection of the Institute of Biotechnology and Genetic Engineering (IBGE), Bangabandhu Sheikh Mujibur Rahman Agricultural University (BSMRAU), Bangladesh. Dry paper discs containing blast fungal isolate were placed in potato dextrose agar, (PDA) containing Petri dishes, and incubated at 25°C for 7 d. A 4 mm freshly grown 7 d old MoT mycelial plug was transferred to Petri dishes containing oatmeal agar (OMA) supplemented with Oracin K (250 mg Phenoxymethylpenicillin; 2 μg per 50 mL OMA, Sanofi Bangladesh Limited) for conidia production and incubated at 25(± 2) °C for 7 d. After 7 d, the Petri dishes were irradiated for 3 d under fluorescent lamps to induce MoT conidiation. To harvest MoT conidia, 10 mL SDW containing 0.02% (V/V) Tween 20 was added to Petri dishes; conidia was harvested from the surface of MoT colony by a sterilized brush; followed by filtering the conidial suspension with sterilized cheesecloth, and the conidial density was adjusted 1 × 10^5^ (Islam et al., 2016) conidia/ml by using a hemocytometer (Fuchs-Rosenthal, 0.0625 mm2) (Gupta et al., 2020).

### 4.3 Antagonism assay

#### 4.3.1 Dual culture assay

A single freshly grown bacterial colony was streaked in PDA (2 cm away from the edge of the Petri dish), and a mycelial plug of MoT (6 mm) from the freshly grown 7 d old culture was placed at the opposite side of Petri dish (9 cm) perpendicular to the bacterial streaks and incubated at 25(±2) °C for 14 d (Zohara et al., 2016). The Petri dish containing only MoT mycelial plugs was used as a control, and five replicates were maintained for each treatment. The percent (%) mycelial growth inhibition was recorded 14 d after inoculation and calculated by the following formula: Mycelial growth inhibition (MGI) % = (C-T/C) × 100; C = growth of MoT in control plate (cm), and T = growth of MoT mycelia in dual cultures (cm).

MoT hyphal morphologies in the vicinity of bacterial colonies were observed under a light microscope (Carl Zeies, Germany), and digital images were recorded by a digital camera (Canon EOS 700D, EF-S 18-55 mm 3.5–5.6 IS STM).

#### 4.3.2 Cell-free culture filtrate assay

A single bacterial colony from each bacterial isolate was incubated in a 100 mL Erlenmeyer flask containing 25 mL potato dextrose broth (PDB) and incubated for 24 h at 28(±2) °C. Then, 100 μL of each bacterial culture was inoculated in 250 mL PDB, in a 500 mL Erlenmeyer flask; incubated at 28(±2) °C for 3 d at 120 rpm. Three days after incubation, the bacterial cells were removed by centrifugation (10,000 rpm for 10 min at 4°C), and 10% bacterial autoclaved (121°C for 60 min) culture filtrates were used for preparing PDA plates. The PDA plates containing Nativo fungicide (60 ppm) were treated as a positive control (most widely used fungicide in Bangladesh to control blast); plates containing only PDA without culture filtrates and fungicide were treated as absolute control. A 6 mm mycelial plug from 7 d old MoT culture was transferred at the center of each Petri dish and incubated at the same conditions described above (2.3.1). The radial MoT hyphal growth in each Petri dish was recorded at 10 d after incubation. Five replicates were used for each treatment, and each experiment was repeated thrice.

### 4.4 Growing of seedlings

The seeds of the wheat BARI Gom-24 was collected from the Bangladesh Agricultural Research Institute, Bangladesh for conducting this study. Wheat seeds cv. BARI Gom–24 (Prodip) was surface sterilized by following the protocol described by Paul et al. (2022). Fifteen seeds were sown in a plastic pot (12 cm × 7.5 cm) containing sterilized field soil amended with NPK Fertilizer and grown for 15 days, maintaining 12/12 h light–dark alternations and 65% humidity. Ten healthy seedlings were allowed to grow under natural conditions, and watering was done as needed.

### 4.5 *In vivo* assay for biocontrol activity of bacterial isolates

Fifteen days after seedling emergence (DAE) two different experiments (preventive and curative) were conducted for seedling assay, and all pots were arranged in a completely randomized design in both experiments. For preventive control assay, 100 mL of each bacterial suspension (*ca.* 1 × 10^8^ (Ceresini et al., 2019) CFU/ml) was sprayed on wheat seedlings at 15 DAE and left 24 h to dry. Twenty-four hours after bacterial inoculation, MoT conidial suspension (ca. 1 × 10^5^ (Islam et al., 2016) conidia/ml) was sprayed on the same wheat seedlings until the plant became wet and covered with polythene bags to maintain humidity (>90%) for 24 h at 25°C in dark conditions to facilitate fungal infection.

For curative control measures, MoT spores (*ca.* 1 × 10^5^ (Islam et al., 2016) conidia/ml) were first spray inoculated in wheat seedlings and covered with polythene bags to maintain humidity (>90%) for 24 h at 25°C in dark to facilitate fungal infection. Twenty four hours after MoT inoculation, the seedlings were sprayed with bacterial suspension (*ca.* 1 × 10^8^ (Ceresini et al., 2019) CFU/ml). Both preventive and curative assays, the seedlings were then transferred to a growth chamber maintaining 25°C temperature, 12 h light per day and > 85% relative humidity. Wheat plants treated without MoT and bacteria served as healthy control, and plants treated with MoT alone without bacterial treatment served as untreated control. Sterilized water was sprayed on inoculated seedlings every day at 4.0 pm to maintain high humidity. Disease development was recorded at 7 d after inoculation, and each treatment was replicated six times (Suryadi et al., 2013; Paul et al., 2022). The disease severity was assessed by six progressive grades from 0 to 5 (Hyon et al., 2012). The scales were: 0 = no lesions; 1 = small, brown, specks of pinhead size; 2 = small, roundish to slightlyelongated necrotic, gray spots about 1–2 mm in diameter; 3 = typical blast lesions infecting <10% of the leaf area; 4 = typical blast lesions infecting 26–50% of the leaf area; and 5 = typical blast lesions infecting >51% of leaf area and many dead leaves. Afterthat, the disease severity was calculated using the following formula:

$\text{DS}\;\left(\%\right)=\frac{\Sigma n\times v}{N\times V}\times 100.$

Where,DS=disease severity.

n=number of leaf infected.

v=value score of each category attack.

N=number of leaves observed.

V=value of highest score

### 4.6 Identification of bacterial isolates

#### 4.6.1 Phenotypic identification of bacterial isolates

Individual pure colonies of selected bacterial isolates grown in the NBA were carefully observed, and colony characteristics-colony type, size, colony color, and shape were recorded (Zohara et al., 2016). A series of physiological and biochemical tests namely-KOH test, gram test, catalase, oxidase, motility test, indole acetic acid (IAA) production, growth in 2, 4, 6, 8 and 10% NaCl were performed for phenotypic characterization of antagonistic bacteria following the methods described by (Paul et al., 2021).

#### 4.6.2 Whole genome sequencing and molecular identification of bacterial isolates

Potential bacterial isolates were further used for molecular identification. The genomic DNA from bacterial isolates was extracted by a commercial GeneJET Genomic DNA extraction kit (Thermo Fisher Scientific, United States). The quality and extracted DNA concentrations were assessed by gel electrophoresis (0.8% agarose) followed by comparing 1 Kb plus DNA ladder (Thermo Fisher Scientific, USA). The genomic DNA was used to construct a whole genome sequence library. The samples were fragmented with Covaris to around 550 to 600 bp, then the NEBNext Ultra DNA library preparation kit for Illumina was used (https://international.neb.com/products/e7370-nebnext-ultra-dna-library-prep-kit-for-llumina#Product%20Information). The constructed libraries were sequenced using Illumina HiSeq platform with 30.0x genome coverage. The raw data was trimmed with Trimmomatic software, and the quality was assessed using in-house scripts combined with Samtools, Bedtools, and bwa-mem softwares. The genome assembly was performed by using SPAdes (v. 3.10.0) method. The assembly matrix was calculated by using “QUAST” software. The taxonomic distribution of bacterial isolates was determined by using Kraken software.

### 4.7 Construction of phylogenetic tree

Genome comparisons and alignments for phylogenetic trees were made using BIGSdb software (Jolley and Maiden, 2010). The digital DNA–DNA hybridization (DDH) were determined online^1^ using the genome to genome distance calculation (GGDC) version 2.0 (Dunlap et al., 2016). The estimated DDH values were calculated using formula two at the GGDC website (Dunlap et al., 2016). Average nucleotide identity (ANI) was calculated with the following options; minimum length 700 bp, minimum identity 70%, minimum alignment 50%, BLASTwindow size 1,000 bp and step size of 200 bp (Dunlap et al., 2016). The alignment used for the phylogenetic tree was based on the core genome of all isolates found in the tree. MEGA X software was used to construct phylogenetic tree (Kumar et al., 2018). Neighbor-joining tree was reconstructed using the Tamura-Nei model (Tamura and Nei, 1993) with a gamma correction (*α* = 0.5) with complete deletion. This model was chosen on the basis of the likelihood test implemented in MEGA X. Measures of bootstrap support for internal branches were obtained from 1,500 pseudoreplicates.

### 4.8 Identification of metabolic genes responsible for antagonism

NCBI Prokaryotic Genome Annotation Pipeline (PGAP) was used for the annotation of predicting protein-coding genes, rRNAs, and tRNAs. Best-placed reference protein set annotation method was used for annotating genome data by using GeneMarkS-2+ software (version 6.1). Rapid Annotation using Subsystems Technology (RAST FIGfams v.70) was used to predict the open reading frames of a genome. The prodigal program was used to predict potential genes in a genome, and the Blastp program was used to find the similarities of predicted proteins against the Uniprot protein database. Metabolic cluster and finding metabolic model was performed by antiMASH software.

### 4.9 Statistical analysis

Statistical data analysis was performed using R software (version 4.1.2). Linear regression models (LMs) were used for analyzing the data sets. The model fit for LMs was evaluated by using “DHARMa” package. The Shapiro–Wilk normality tests (shapiro.test function) was performed to determine whether the response variables met test assumptions. Analysis of variance (ANOVA) followed by Tukey multiple comparison (*p* ≤ 0.05) was performed using “emmeans” package for normally distributed data and Kruskal-Wallis test using “kruskal.test” function followed by Dunn multiple comparison analysis using “FSA” and “rcompanion” packages (*p* < 0.05) for non-normally distributed data sets. The plots were visualized by using “ggplot2” function.

All experimental methods described above were carried out in accordance with relevant guidelines and regulations.

## 5 Conclusion

Three seed bacteria isolates viz. *B. velezensis* (BTS-4 and BTKL6A) and *B. subtilis* (BTS-3) were identified and characterized as potential candidates for biological control of wheat blast through laboratory and greenhouse assays. The whole-genome sequence data revealed that these three bacterial isolates contain different antimicrobial, cell wall degrading, induced systemic resistance and antioxidant enzyme-related gene clusters which potentially play an important role in antagonism and suppression of MoT growth in host plants. Further studies are needed to precisely identify the specific genes involved in the production of antimicrobial substances and also characterize the structural features of the chemical arsenals produced by those bacterial isolates. A large-scale field evaluation of the efficacy of biocontrol bacteria at the reproductive stages of wheat is needed before recommending them for practical use as biocontrol agents against wheat blast fungus in the practical field.

## Data availability statement

The datasets presented in this study can be found in online repositories. The names of the repository/repositories and accession number(s) can be found in the article/Supplementary material.

## Author contributions

MS: Investigation, Writing – original draft, Formal analysis, Investigation. SD: Formal analysis, Software, Visualization, Writing – original draft. NM: Investigation, Writing – original draft. DG: Data curation, Writing – review & editing. TF: Investigation, Methodology, Writing – review & editing. SP: Investigation, Visualization, Writing – review & editing. JW: Formal analysis, Investigation, Writing – review & editing. CD: Formal analysis, Software, Writing – review & editing. RO: Formal analysis, Writing – review & editing. MR: Validation, Writing – review & editing. AS: Data curation, Writing – review & editing. TI: Conceptualization, Funding acquisition, Project administration, Supervision.

## Footnotes

^1^ https://ggdc.dsmz.de/distcalc2.php
