# Supplementary material for: Biological control potential of worrisome wheat blast disease by the seed endophytic bacilli
Source: Front Microbiol. 2024 Mar 11;15:1336515. doi: 10.3389/fmicb.2024.1336515 (PMC10961374; doi:10.3389/fmicb.2024.1336515)
Supplement: Supplementary file 1 [file Table_1.DOCX]

**Supplementary table 1: List of bacterial isolates used in dual culture assay against *Magnaporthe oryzae* pathotype *Triticum***

| **Serial no.** | **Bacterial isolates** | **Source** | **Mycelial inhibition (%)** |
| --- | --- | --- | --- |
| 1 | BSm1 | Shatamuli | 25.3 |
| 2 | BCh1 | Chilli | 5.0 |
| 3 | BAr2 | Arjun | 15.0 |
| 4 | BDs1 | Drum stick | 20.0 |
| 5 | BKh1 | Khayer | 6.0 |
| 6 | BThL | Thankuni leaf | 5.4 |
| 7 | BThR | Thankuni root | 5.45 |
| 8 | BPlc1 | Palm tree | 15.75 |
| 9 | BSng1 | Snake plant | 17.57 |
| 10 | BNpl3 | Nayantara leaf | 20.48 |
| 11 | BTL001 | Brinjal leaf | 16.51 |
| 12 | BTL002 | Ulu leaf | 1.50 |
| 13 | BTL003 | Rice seed | 17.44 |
| 14 | BTL004 | Tishi leaf | 16.38 |
| 15 | BTL005 | Foska begun leaf | 19.29 |
| 16 | BTL006 | Durba grass | 12.9 |
| 17 | BTL007 |  | 14.81 |
| 18 | BTL008 | Lemon | 15.6 |
| 19 | BTLbbc-01 | Chilli | 27.56 |
| 20 | BTLbbc-02 |  | 12.66 |
| 21 | BTLbbc-03 |  | 8.67 |
| 22 | BTLbbc-04 |  | 21.0 |
| 23 | BTLbbc-05 |  | 5.33 |
| 24 | BTLbbc-06 |  | 8.97 |
| 25 | BTLbbc-07 |  | 17.67 |
| 26 | Bph-4 | Soil | 9.78 |
| 27 | Bph-100 |  | 14.8 |
| 28 | Bsb-Z |  | 17.3 |
| 29 | Bsb-8 |  | 19.21 |
| 30 | Bsb-101 |  | 16.3 |
| 31 | Bph-6 |  | 17.8 |
| 32 | Bsb-106 |  | 26.5 |
| 33 | PPB 1 | Maize | 19.81 |
| 34 | PPB 2 | Bermuda grass | 27.44 |
| 35 | PPB 3 | Torpedo grass | 17.12 |
| 36 | PPB 4 | Bottle gourd | 37.79 |
| 37 | PPB 5 | Cucumber | 11.34 |
| 38 | PPB 6 | Torpedo grass | 18.32 |
| 39 | PPB 7 | Cucumber | 24.66 |
| 40 | PPB 8 |  | 6.65 |
| 41 | PPB 9 |  | 14.33 |
| 42 | PPB 10 | Bottle gourd | 29.74 |
| 43 | PPB 11 | Cucumber | 18.51 |
| 44 | PPB 12 |  | 26.15 |
| 45 | BCL-1 | Cassava leaf | 16.11 |
| 46 | BRtL-2 | Ram tulsi leaf | 14.67 |
| 47 | BDR-2 | Duranta root | 15.08 |
| 48 | BBgL-1 | Valvet apple leaf | 18.89 |
| 49 | BBgL-2 |  | 0.0 |
| 50 | BBgL-3 |  | 1.13 |
| 51 | BGL-1 | Grape leaf | 8.01 |
| 52 | BGL-2 |  | 1.01 |
| 53 | BDR-1 | Duranta root | 0.0 |
| 54 | BDL-1 | Duranta leaf | 0.0 |
| 55 | BDL-2 |  | 2.24 |
| 56 | BRtL-1 | Ram tulsi leaf | 1.76 |
| 57 | BRtL-3 |  | 9.15 |
| 58 | BBoS-1 | Bohera seed | 0 |
| 59 | BCL-2 | Cassava leaf | 12.40 |
| 60 | BCL-3 |  |  |
| 61 | BTLTW1 | Wheat (Kheri) | 12.72 |
| 62 | BTLTW2 | Wheat (Kalyansona) | 16.33 |
| 63 | BTLTW3 | Wheat (Durum) | 0.0 |
| 64 | BTLTW4 |  | 1.07 |
| 65 | BTLTW5 |  | 13.3 |
| 66 | BTLTW6 |  | 0.0 |
| 67 | BTLTW7 | Wheat (Triticale) | 2.70 |
| 68 | BTLTW8 |  | 17.0 |
| 69 | BTLTW9 |  | 13.3 |
| 70 | BTLTW10 | Wheat (Bijoy) | 0.0 |
| 71 | BTLTW11 | Wheat (Kanchan) | 5.73 |
| 72 | BTLTW12 | Wheat (BARI Wheat 23) | 19.5 |
| 73 | BTLK6ap | Wheat (Kanchan) | 15.16 |
| 74 | BTLK6A |  | 53.16 |
| 75 | BTLK6b |  | 0.0 |
| 76 | BTLK6c |  | 11.16 |
| 77 | BTLK6D |  | 0.0 |
| 78 | BTLK6e |  | 0.0 |
| 79 | BTLK6f |  | 1.9 |
| 80 | BTLK6Y |  | 3.67 |
| 81 | BTLP6A | Wheat (Pradip) | 4.16 |
| 82 | BTLP6b |  | 12.05 |
| 83 | BTLP6c |  | 13.5 |
| 84 | BTL23A | Wheat (BARI Wheat 23) | 3.33 |
| 85 | BTL23b |  | 3.06 |
| 86 | BTL23c |  | 11.67 |
| 87 | BTL25a | Wheat (BARI Wheat 25) | 12.0 |
| 88 | BTL25b |  | 6.53 |
| 89 | BTL28a | Wheat (BARI Wheat 28) | 5.97 |
| 90 | BTL28b |  | 8.05 |
| 91 | BTLD1 | Wheat (Durum) | 2.32 |
| 92 | BTLD2 |  | 1.9 |
| 93 | BTLD3 |  | 3.21 |
| 94 | BTLD4 |  | 0.0 |
| 95 | BTLT1 | Wheat (Triticale) | 0.0 |
| 96 | BTLT2 |  | 7.13 |
| 97 | BTLSu1 | Wheat (Sufi) | 0.0 |
| 98 | BTLSu2 |  | 0.0 |
| 99 | BTLSu3 |  | 0.0 |
| 100 | BTLSu4 |  | 0.0 |
| 101 | BTLBi1 | Wheat (Bijoy) | 1.72 |
| 102 | BTLBi2 |  | 31.7 |
| 103 | BTLBi3 |  | 11.1 |
| 104 | BTLKh1 | Wheat (Kheri) | 17.6 |
| 105 | BTLKh2 |  | 16.4 |
| 106 | BTLKh3 |  | 37.6 |
| 107 | BTLKh4 |  | 5.86 |
| 108 | BTLKh5 |  | 9.93 |
| 109 | BTLSon1 | Wheat (Sonora) | 0.0 |
| 110 | BTLSon2 |  | 0.0 |
| 111 | BTLSon3 |  | 0.0 |
| 112 | BTLPt1 | Wheat (Protiva) | 34.6 |
| 113 | BTLPt2 |  | 30.07 |
| 114 | BTLPt3 |  | 35.7 |
| 115 | BTLPt4 |  | 21.6 |
| 116 | BTLAg1 | Wheat (Aghrani) | 37.5 |
| 117 | BTLAg2 |  | 13.4 |
| 118 | BTLAg3 |  | 27.5 |
| 119 | BTLAg4 |  | 23.6 |
| 120 | BTLL1 | Rice (Lalzira) | 12.09 |
| 121 | BTLL6 |  | 19.76 |
| 122 | BTLN7 | Rice (Nunia) | 0.0 |
| 123 | BTLN10 |  | 14.33 |
| 124 | BTLJ11 | Rice (Joyna) | 7.50 |
| 125 | BTLC12 | Rice (Chinikanai) | 0.0 |
| 126 | BTLK13 | Rice (Kartikbalam) | 0.0 |
| 127 | BTLK18 |  | 0.0 |
| 128 | BTLD19 | Rice (Dursar) | 24.93 |
| 129 | BTLD20 |  | 17.10 |
| 130 | BTLD21 |  | 10.63 |
| 131 | BTLD22 |  | 17.13 |
| 132 | BTLD23 |  | 18.01 |
| 133 | BTLK24 | Rice (Kalozira) | 8.3 |
| 134 | BTLK25 |  | 24.14 |
| 135 | BTLK26 |  | 15 |
| 136 | BTLK27 |  | 7.13 |
| 137 | BTLK28 |  | 17.04 |
| 138 | BTLK29 |  | 11.3 |
| 139 | BTLK30 |  | 6.32 |
| 140 | BTLG31 | Rice (Gore Kajal) | 17.25 |
| 141 | BTLG32 |  | 15.03 |
| 142 | BTLG33 |  | 8.38 |
| 143 | BTLG34 |  | 18.08 |
| 144 | BTLG35 |  | 17.11 |
| 145 | BTLG36 |  | 0.0 |
| 146 | BTLG37 |  | 18.96 |
| 147 | BTLG38 |  | 0.0 |
| 148 | BTLS39 | Rice (Sadamata) | 0.0 |
| 149 | BTLS40 |  | 18.22 |
| 150 | BTLS41 |  | 14.83 |
| 151 | BTLS42 |  | 7.19 |
| 152 | BTLM43 | Rice (Malsira) | 0.0 |
| 153 | BTLM44 |  | 18.47 |
| 154 | BTLM45 |  | 17.56 |
| 155 | BNA6 | Rice (BINA-14) | 5.73 |
| 156 | BTLB48 | Rice (Bashmoti) | 8.67 |
| 157 | B49 |  | 12.15 |
| 158 | BTLA46 | Rice (Aijong) | 0.0 |
| 159 | BTS-1 | Rice (Bashmoti) | 36.12 |
| 160 | BTS-2 | Rice (Moulata) | 37.07 |
| 161 | BTS-3 | Rice (Rangabinni) | 50.71 |
| 162 | BTS-4 |  | 49.42 |
| 163 | BTS-5 | Rice (Malshira) | 41.4 |
| 164 | BTS-6 | Rice (Lalzira) | 6.13 |
| 165 | BTS-8 |  | 24.89 |
| 166 | BTS-9 |  | 26.2 |
| 167 | BTS-7 | Rice (Moulata) | 0.0 |
| 168 | BTS-10 | Rice (Darshail) | 38.7 |
| 169 | BTS-13 | Rice (Shakhorkora) | 28.4 |
| 170 | BTS-14 |  | 15.76 |

**Supplementary table 2: List of bacterial isolates used for phylogenetic analysis**

| **Sl.** | **Bacterial isolates** | **BioProject** | **Accession** | **Genome size (Mb)** | **Reference** |
| --- | --- | --- | --- | --- | --- |
| 1 | *Bacillus inaquosorum* KCTC 13429 | PRJNA224116 | AMXN01 | 4.3 | Yi H, et al. Genomic insights into the taxonomic status of the three subspecies of *Bacillus subtilis*. Syst Appl Microbiol. 2014. 37(2):95-9. |
| 2 | *B. spizizenii* str TU-B-10 | PRJNA68561 | CP002905 | 4.2 | Earl AM, et al. Whole-genome sequences of *Bacillus subtilis* and close relatives. J Bacteriol. 2012. 194(9):2378-9 |
| 3 | *B. subtilis* NCIB 3610 | PRJNA377766 | CP020102 | 4.2 | Nye T, et al. *Bacillus subtilis* strain NCIB 3610. Unpublished |
| 4 | *B. tequilensis* KCTC 13622 | PRJNA224116 | AYTO01 | 4.2 | Jeong H, et al. Draft genome sequence of *Bacillus tequilensis* 13622^T^. Unpublished |
| 5 | *B. cabrialesii* TE3 | PRJNA224116 | RJVS01 | 4.1 | de Los Santos Villalobos S, et al. *Bacillus cabrialesii* sp. nov., an endophytic plant growth promoting bacterium isolated from wheat (*Triticum turgidum* subsp. *durum*) in the Yaqui Valley, Mexico. Int J Syst Evol Microbiol. 2019 69(12):3939-3945. |
| 6 | *B. vallismortis* DV1-F-3 | PRJNA68565 | AFSH01 |  | Earl AM, et al.  Whole genome sequences of *Bacillus subtilis* and close relatives. Unpublished |
| 7 | *B. mojavensis* KCTC 3706 | PRJNA224116 | AFS101 | 4.1 | Jeong H, et al. Draft genome sequence of *Bacillus mojavensis* KCTC 3706. Unpublished |
| 8 | *B. halotolerans* ATCC 25096 | PRJNA224116 | LPVF01 | 4.2 | Dunlap CA, et al. Genome analysis shows *Bacillus axarquiensis* is not a later heterotypic synonym of *Bacillus mojavensis*; reclassification of *Bacillus malacitensis* and *Brevibacterium halotolerans* as heterotypic synonyms of *Bacillus axarquiensis*. Int J Syst Evol Microbiol. 2016. 66(6):2438-2443. |
| 9 | *B. atrophaeus* NRRL NRS 213 | PRJNA224116 | LSBB01 | 4.2 | Dunlap C. *Bacillus atrophaeus*. Unpublished |
| 10 | *B. nakamurai* NRRL B-41091 | PRJNA224116 | LSAZ01 | 3.9 | Dunlap C. *Bacillus nakamurai.* Unpublished |
| 11 | *B. amyloliquefaciens* DSM7 | PRJEA41719 | FN597644 | 3.9 | Borriss R, et al. Relationship of *Bacillus amyloliquefaciens* clades associated with strains DSM 7T and FZB42T: a proposal for *Bacillus amyloliquefaciens* subsp. *amyloliquefaciens* subsp. nov. and *Bacillus amyloliquefaciens* subsp. *plantarum* subsp. nov. based on complete genome sequence comparisons. Int J Syst Evol Microbiol. 2011. 61(Pt 8):1786-1801. |
| 12 | *B. siamensis* KCTC 13613^T^ | PRJNA224116 | AJVF01 | 3.9 | Jeong H, et al. Draft genome sequence of the plant growth-promoting bacterium *Bacillus siamensis* KCTC 13613T. J Bacteriol. 2012. 194(15):4148-9. |
| 13 | *B. velezensis* NRRL B-41580 | PRJNA224116 | LLZC0 | 4.0 | Dunlap CA, et al. *Bacillus velezensis* is not a later heterotypic synonym of *Bacillus amyloliquefaciens*; *Bacillus methylotrophicus, Bacillus amyloliquefaciens* subsp. *plantarum* and *'Bacillus oryzicola*' are later heterotypic synonyms of *Bacillus velezensis* based on phylogenomics. Int J Syst Evol Microbiol. 2016. 66(3):1212-1217. |
| 14 | *B. licheniformis* DSM 13 | PRJNA12388 | CP000002 | 4.2 | Rey MW, et al. Complete genome sequence of the industrial bacterium *Bacillus licheniformis* and comparisons with closely related Bacillus species. Genome Biol. 2004. 5(10):R77. |
| 15 | *B. paralicheniformis* KJ-16 | PRJNA224116 | LBMN02 | 4.6 | Dunlap CA, et al. *Bacillus paralicheniformis* sp. nov., isolated from fermented soybean paste. Int J Syst Evol Microbiol. 2015. 65(10):3487-3492. |
| 16 | *B. haynesii* B-41327 | PRJNA224116 | MRBL01 | 4.4 | Dunlap CA, et al. *Bacillus swezeyi* sp. nov. and *Bacillus haynesii* sp. nov., isolated from desert soil. Int J Syst Evol Microbiol. 2017. 67(8):2720-2725. |
| 17 | *B. swezeyi* B-41294 | PRJNA224116 | MRBK01 | 4.5 | Dunlap CA, et al. *Bacillus swezeyi* sp. nov. and Bacillus haynesii sp. nov., isolated from desert soil. Int J Syst Evol Microbiol. 2017. 67(8):2720-2725. |
| 18 | *B. glycinifermentans* GO-13^T^ | PRJNA224116 | LECW01 | 4.4 | Zeigler DR. Genome Sequence of *Bacillus glycinifermentans* TH008, isolated from Ohio Soil. Genome Announc. 2016. 21;4(1):e01573-15. |
| 19 | *B. sonorensis* KCTC 13918 | PRJNA227788 | AYTN01 | 4.4 | Jeong H, et al. Draft genome sequence of *Bacillus sonorensis* 13918T. Unpublished |
